# Wiskott-Aldrich综合征并滤泡性淋巴瘤1例

**DOI:** 10.3760/cma.j.cn121090-20251204-00568

**Published:** 2026-07

**Authors:** 莹 张, 岩 张, 启燕 陈, 书杰 王, 明辉 段, 薇 张

**Affiliations:** 1 北京市隆福医院血液科，北京 100010 Department of Hematology, Beijing Longfu Hospital, Beijing 100010, China; 2 北京协和医院血液科，北京 100730 Department of Hematology, Peking Union Medical College Hospital, Beijing 100730, China

患者，男，22岁。2025年2月无意中发现颈部双侧多发肿大淋巴结，无痛性进行性增大。2025年3月就诊于北京协和医院血液科门诊，血常规：PLT 27×10^9^/L［参考值（125～350）×10^9^/L］，平均血小板体积（MPV）4.8 fl（参考值7.0～11.0 fl）。免疫固定电泳：免疫球蛋白（Ig）G λ型M蛋白阳性。血清蛋白电泳：M蛋白百分比5.4％、浓度3.26 g/L。颈部淋巴结活检病理诊断：滤泡性淋巴瘤，3A级。2025年5月于北京市隆福医院血液科住院治疗，骨髓形态：符合血小板减少症。骨髓活检及流式细胞术未见淋巴瘤细胞浸润。染色体正常核型。PET-CT示全身多组淋巴结肿大伴代谢异常增高，SUVmax为14.3，多处淋巴结病灶直径>3.0 cm，符合淋巴瘤影像学表现。2025年5月14日开始予G-CHOP方案化疗6个疗程，具体方案：奥妥珠单抗1 000 mg静脉输注，第0天；环磷酰胺1.4 g+多柔比星130 mg+长春地辛4 mg静脉输注，第1天；泼尼松100 mg口服，第1～5天；21 d为1个疗程。第4个疗程化疗后复查PET-CT，为代谢完全缓解（CR）。治疗期间患者反复出现细菌、病毒及肺孢子菌肺炎感染。PLT波动于（49～74）×10^9^/L，阿伐曲波帕治疗后逐渐升高，第5个疗程后恢复正常，但MPV仍持续偏低（5.2～6.8 fL）。既往史：患者自2岁起反复出现湿疹，皮肤瘀点、瘀斑及鼻衄，PLT最低至25×10^9^/L，当时未发现淋巴结肿大，曾行骨髓穿刺检查（具体不详），诊断为“特发性血小板减少症”，经泼尼松、静脉注射免疫球蛋白治疗效果不佳，监测血小板波动于（30～40）×10^9^/L，间断出血倾向，可自行缓解。后行WAS基因全外显子组测序，提示内含子6存在c.559+5G>A半合子变异。诊断：Wiskott-Aldrich综合征合并滤泡淋巴瘤。

讨论：Wiskott-Aldrich综合征是WAS基因突变导致WAS蛋白（WASP）表达缺陷所致的原发性免疫缺陷病，典型三联征为湿疹、血小板减少和反复感染，其罹患自身免疫性疾病和恶性肿瘤的风险显著增加。本例患者自幼年起病，持续性血小板减少伴MPV显著减低，是提示WAS诊断的重要线索。血液和淋巴系统的恶性肿瘤是WAS的并发症，但合并滤泡性淋巴瘤的病例较少。患者根据Lugano分期属于Ⅲ期，血常规异常。IgGλ型M蛋白阳性；淋巴结病免疫组化结果显示CD5阳性，提示其侵袭性更高，向弥漫大B细胞淋巴瘤转化率更高。治疗需同时应对两方面挑战：一是有效控制恶性肿瘤，二是基础免疫缺陷带来的出血与感染风险。本例患者采用G-CHOP方案作为一线化疗，旨在高效清除肿瘤细胞；同时，自化疗前便全程应用血小板生成素受体激动剂（TPO-RA），以纠正严重的血小板减少，为化疗的顺利进行提供了关键保障。本例患者临床表现为轻型，虽经TPO-RA治疗PLT可恢复正常，G-CHOP方案治疗后达到CR，但Wiskott-Aldrich综合征若不根治，患者仍持续处于继发其他恶性肿瘤的高风险状态，因此建议其亲属行全基因组测序检查，并尽早行异基因造血干细胞移植治疗，实现完全治愈。

